# Genetic Association Analysis of *NOS1* and Methamphetamine-Induced Psychosis Among Japanese

**DOI:** 10.2174/157015911795017308

**Published:** 2011-03

**Authors:** Takenori Okumura, Tomo Okochi, Taro Kishi, Masashi Ikeda, Tsuyoshi Kitajima, Yoko Kinoshita, Kunihiro Kawashima, Tomoko Tsunoka, Yasuhisa Fukuo, Toshiya Inada, Mitsuhiko Yamada, Naohisa Uchimura, Masaomi Iyo, Ichiro Sora, Norio Ozaki, Hiroshi Ujike, Nakao Iwata

**Affiliations:** 1 Department of Psychiatry, Fujita Health University School of Medicine, Toyoake, Japan; 2 Department of Psychiatry, Teikyo University Ichihara Hospital, Ichihara, Japan; 3 Department of Psychogeriatrics, National Institute of Mental Health, National Center of Neurology and Psychiatry, Kodaira, Japan; 4 Department of Neuropsychiatry, Kurume University Graduate School of Medicine, Kurume, Japan; 5 Department of Psychiatry, Chiba University Graduate School of Medicine, Chiba, Japan; 6 Department of Psychobiology, Tohoku University Graduate School of Medicine, Sendai, Japan; 7 Department of Psychiatry and Psychobiology, Nagoya University Graduate School of Medicine, Nagoya, Japan; 8 Department of Neuropsychiatry, Okayama University Graduate School of Medicine, Dentistry and Pharmaceutical Science, Okayama, Japan; 9 Japanese Genetics Initiative for Drug Abuse (JGIDA)

**Keywords:** Methamphetamine-induced psychosis, neuronal nitric oxide synthase 1 gene (*NOS1*), case-control association study.

## Abstract

The neuronal nitric oxide synthase gene (*NOS1*) is located at 12q24, a susceptibility region for schizophrenia, and produces nitric oxide (NO). NO has been reported to play important roles as a gaseous neurotransmitter in brain. NO is a second messenger for the N-methyl-D aspartate (NMDA) receptor and is related to the dopaminergic system. Because the symptomatology of methamphetamine (METH) use disorder patients with psychosis is similar to that of patients with schizophrenia, *NOS1* is a good candidate gene for METH-induced psychosis. Therefore, we conducted a case-control association study between *NOS1* and METH-induced psychosis with Japanese subjects (183 with METH-induced psychosis patients and 519 controls). We selected seven SNPs (rs41279104, rs3782221, rs3782219, rs561712, rs3782206, rs6490121, rs2682826) in *NOS1* from previous reports. Written informed consent was obtained from each subject. This study was approved by the Ethics Committee at Fujita Health University School of Medicine and each participating institute of the Japanese Genetics Initiative for Drug Abuse (JGIDA). No significant association was found between *NOS1* and METH-induced psychosis in the allele/genotype-wise or haplotype-wise analyses. In conclusion, we suggest that *NOS1* might not contribute to the risk of METH-induced psychosis in the Japanese population.

## 1 INTRODUCTION

Methamphetamine (METH) is a drug that is used widely in the world and causes psychiatric disorder. METH causes abnormalities of the dopamine neural transmission in the mesolimbic system, and is thought to cause psychotic symptoms such as hallucinations and delusions [1, 2].

Nitric oxide (NO) is involved a variety of mechanisms, regulating the release of neurotransmitters such as dopamine and serotonin, activating N-methyl-D-aspartate (NMDA) receptor, and participating in oxidative stress [3-5]. Therefore, NO functions may be considered to induce psychotic disorders. The nitric oxide synthase 1 gene (*NOS1*) is a complex gene located at 12q24, consisting of 12 alternative untranslated first exons, termed exon 1a_1l, and 28 exons in a genomic region spanning 149.404Kb. *NOS1* is considered to be a likely candidate gene for schizophrenia owing to its chromosomal location, 12q24, which has been reported to be a susceptibility locus from several linkage studies, and to play a role in producing NO in the human brain [6-8]. 

Several genetic association studies showed that single nucleotide polymorphisms (SNPs) in *NOS1* were associated with schizophrenia. Reif *et al.* identified functional SNP (rs41279104) in the promoter region and found an association with schizophrenia [9]. Two other genetic association studies showed a significant association between rs2682826 in exon 29 and haplotype (rs3782221-rs3782219-rs561712-rs3782206) and schizophrenia. Recently, a whole genome association study reported an association between rs6490121 in intron 2 of *NOS1* and schizophrenia [12]. Therefore, *NOS1* is recognized to be candidate gene for schizophrenia [10, 11].

Because the symptoms of METH-induced psychosis are similar to those of paranoid type schizophrenia, it may be that the METH-induced psychosis and schizophrenia have common susceptibility genes. Therefore, it would be of interest to examine the association between *NOS1* and METH-induced psychosis. We conducted a genetic association analysis in the Japanese population. 

## 2 MATERIALS AND METHODS

### 2.1 Subjects

The subjects in the association analysis were 183 patients (all patients were diagnosed as having METH-induced psychosis; 151 males and 32 females: mean age ± SD 36.7±11.6 years) and 519 healthy controls (268 males and 251 females: mean age ± SD 37.5 ±14.4 years). All subjects were unrelated to each other, ethnically Japanese, and lived in Japan. Among the subjects with METH use disorder, all subjects had a comorbid diagnosis of METH-induced psychosis. One hundred forty-nine subjects with METH use disorder abused or had dependence on drugs other than METH. Cannabinoids were the most frequency abused drugs (31.4%), followed by cocaine (9.09%), LSD (9.09%), opioids (7.69%), and hypnotics (7.69%). Subjects with METH use disorder were excluded if they had a clinical diagnosis of psychotic disorder, mood disorder, anxiety disorder, or eating disorder. The patients were diagnosed according to DSM-IV or ICD-10 criteria with consensus of at least two experienced psychiatrists on the basis of unstructured interviews and a review of medical records. All healthy controls were also psychiatrically screened through unstructured interviews, and those with past individual or family history of drug dependence or an axis 1 disorder such as psychotic or mood disorder were excluded. After describing the study, written informed consent was obtained from each subject. This study was approved by the Ethics Committee at Fujita Health University and each participating institute of the Japanese Genetics Initiative for Drug Abuse (JGIDA).

2.2

SNP Selection and Genotyping

We selected seven SNPs (rs2682826, rs3782221, rs3782219, rs561712, rs3782206, rs41279104, rs6490121) in *NOS1* from previous association studies for schizophrenia [9-11]. We used TaqMan assays (Applied Biosystems) for all SNPs. 

### 2.3 Statistical Analysis

Genotype deviation from the Hardy-Weinberg equilibrium (HWE) was evaluated with the chi-square test (SAS/Genetics, release 8.2, SAS Japan INC, Tokyo, Japan). Marker-trait association was also evaluated with the chi-square test in allele- and genotype-wise analyses. Haplotype frequencies were estimated in a two- to four-marker sliding window fashion and log likelihood ratio tests were performed for global P-values with COCAPHASE program version 3.0.6 [13]. In these haplotype-wise analyses, rare haplotypes (less than 0.05) of either cases or controls were excluded from the association analysis. Power calculation was performed using a statistical program prepared with the Genetic Power Calculator (http://pngu.mgh.harvard.edu-/~purecell//gpc/). The level of significance for all statistical tests was 0.05.

## 3 RESULTS

Genotype frequencies of subjects and controls did not deviate significantly from HWE. No significant association was found between *NOS1* and METH-induced psychosis in the allele/genotype-wise analysis, or in the haplotype analysis (Table **1**, **2**). Our METH samples were unmatched gender samples for METH-induced psychosis. Therefore, we performed an explorative analysis of gender effects, but no association was detected between any of the SNPs and either sex (Table **3**).

In a power analysis, we obtained more than 80% power for the detection of association when we set the genotype relative risk at 1.37-1.6, under a multiplicative model of inheritance.

## 4 DISCUSSION

We did not find an association between the seven SNPs in *NOS1* and METH-induced psychosis in the allele/genotype-wise or haplotype-wise analysis in these subjects. In several genetic studies of METH, gender effects were found in METH use disorder [14, 15]. Because we recognize that our gender samples were unmatched, our negative result may have mainly reflected the effect of male METH-induced psychosis. We therefore conducted an explorative analysis of gender effects, but found none. Since NO has an important role in regulating the release of neurotransmitters such as dopamine and serotonin, *NOS1* is recognized to be a good candidate gene for disorder with psychosis. However, a previous study did not report an association between *NOS1* and schizophrenia [16]. Other genes involved in the activity of *NOS1* may be related to the pathophysiology of psychotic disorders such as schizophrenia and METH-induced psychosis.

Nitric oxide synthase 1 adaptor protein (*NOS1AP*) encodes an adapter protein that binds to *NOS1 *and links to a specific target. Recent studies reported evidence of a significant association between *NOS1AP* and schizophrenia [17, 18]. Considering these positive results, it will be necessary to replicate the studies using other larger population samples and other phenotypes, such METH-induced psychosis.

A few points of caution must be mentioned with regard to our present negative findings. (1) It is important to evaluate associations between METH use disorder with and without psychosis. However, since we had only a small number of subjects without psychosis, we did not evaluate this association to avoid type I error due to small sample size. to the small number of subjects was a result of limitations of sample collection, since we recruited cases of METH use disorder in psychiatric hospitals. (2) We could not adopt an LD-based strategy and mutation scan, because *NOS1* has a massive gene structure. Therefore, in future studies it will be necessary to evaluate associations between other common variants or rare variants with functional effects and *NOS1* in METH use disorder. 

In conclusion, our results suggest that *NOS1* does not play a major role in METH use disorder with psychosis in the Japanese population. However, the number of METH samples used in this study was small, and even though it is difficult to find samples of METH use disorder, it will be necessary to validate or replicate our association in other, larger population samples. 

## Figures and Tables

**Table 1:** Association Study Between *NOS1* and METH-Induced Psychosis

SNP ID	Phenotype^a^	MAF^b^	N	Genotype Distribution^c^			P-Value		
				M/M	M/m	m/m	HWE^d^	Genotype	Allele
rs41279104	METH-induced psychosis	0.175	183	123	56	4	0.413	0.684	0.983
	CON	0.175	519	354	148	17	0.751		
rs3782221	METH-induced psychosis	0.421	183	61	90	32	0.903	0.912	0.891
	CON	0.425	519	175	247	97	0.55		
rs3782219	METH-induced psychosis	0.423	183	61	89	33	0.956	0.611	0.332
	CON	0.453	519	154	260	105	0.803		
rs561712	METH-induced psychosis	0.161	183	128	51	4	0.679	0.716	0.435
	CON	0.179	519	346	160	13	0.274		
rs3782206	METH-induced psychosis	0.265	183	100	69	14	0.663	0.745	0.44
	CON	0.245	519	300	184	35	0.351		
rs6490121	METH-induced psychosis	0.407	183	62	93	28	0.475	0.523	0.532
	CON	0.426	519	175	246	98	0.484		
rs2682826	METH-induced psychosis	0.380	183	67	93	23	0.286	0.175	0.0754
	CON	0.329	519	230	237	52	0.424		

a METH:methamphetamine CON: control

b MAF: minor allele frequency

c M: major allele, m: minor allele

d Hardy-Weinberg equilibrium.

**Table 2:** Haplotype-Wise Analysis Between *NOS1* and METH-Induced Psychosis

	Global *P*-value		
SNP ID	2 Window	3 Window	4 Window
rs41279104			
	0.631		
rs3782221		0.576	
	0.336		0.755
rs3782219		0.541	
	0.385		0.799^a^
rs561712		0.683	
	0.756		0.673
rs3782206		0.810	
	0.428		0.871
rs6490121		0.868	
	0.769		
rs2682826			

a Fallin *et al.* reported

**Table 3:** Results of Explorative Analysis Between *NOS1* and METH-Induced Psychosis

SNP ID	Gender	Phenotype^a^	MAF b	N	Genotype Distribution c			P-Value		
					M/M	M/m	m/m	HWE d	Genotype	Allele
rs41279104	male	METH-induced psychosis	0.172	151	102	46	3	0.399	0.977	0.853
		CON	0.177	268	179	83	6	0.310		
	female	METH-induced psychosis	0.187	32	21	10	1	0.884	0.597	0.452
		CON	0.151	251	184	58	9	0.110		
rs3782221	male	METH-induced psychosis	0.423	151	50	74	27	0.966	0.988	0.882
		CON	0.429	268	87	132	49	0.931		
	female	METH-induced psychosis	0.453	32	7	21	4	0.0667	0.106	0.574
		CON	0.416	251	89	115	47	0.364		
rs3782219	male	METH-induced psychosis	0.423	151	50	74	27	0.966	0.541	0.326
		CON	0.458	268	75	140	53	0.395		
	female	METH-induced psychosis	0.515	32	8	15	9	0.727	0.572	0.293
		CON	0.446	251	79	120	52	0.605		
rs561712	male	METH-induced psychosis	0.188	151	100	45	6	0.741	0.253	0.119
		CON	0.147	268	194	69	5	0.689		
	female	METH-induced psychosis	0.141	32	23	9	0	0.354	0.343	0.175
		CON	0.213	251	152	91	8	0.200		
rs3782206	male	METH-induced psychosis	0.264	151	82	58	11	0.865	0.956	0.860
		CON	0.259	268	149	99	20	0.529		
	female	METH-induced psychosis	0.296	32	17	11	4	0.317	0.243	0.174
		CON	0.221	251	153	85	13	0.789		
rs6490121	male	METH-induced psychosis	0.417	151	53	70	28	0.565	0.182	0.0736
		CON	0.481	268	72	134	62	0.981		
	female	METH-induced psychosis	0.375	32	13	14	5	0.706	0.281	0.0937
		CON	0.486	251	73	112	66	0.0904		
rs2682826	male	METH-induced psychosis	0.403	151	50	80	21	0.218	0.360	0.935
		CON	0.401	268	99	123	46	0.464		
	female	METH-induced psychosis	0.515	32	9	13	10	0.290	0.190	0.0952
		CON	0.406	251	92	114	45	0.352		

a METH:methamphetamine CON:control

b MAF: minor allele frequency

c M: major allele, m: minor allele

d Hardy-Weinberg equilibrium
